# Alleviating effects of coenzyme Q10 supplements on biomarkers of inflammation and oxidative stress: results from an umbrella meta-analysis

**DOI:** 10.3389/fphar.2023.1191290

**Published:** 2023-08-08

**Authors:** Sara Dabbaghi Varnousfaderani, Vali Musazadeh, Faezeh Ghalichi, Zeynab Kavyani, Soha Razmjouei, Amir Hossein Faghfouri, Sana Sedgh Ahrabi, Seyyed Morteza Seyyed Shoura, Parvin Dehghan

**Affiliations:** ^1^ Department of Pharmacy, Shahid Beheshti University of Medical Science, Tehran, Iran; ^2^ Student Research Committee, Tabriz University of Medical Sciences, Tabriz, Iran; ^3^ School of Nutrition and Food Sciences, Tabriz University of Medical Sciences, Tabriz, Iran; ^4^ Nutrition Research Center, Faculty of Nutrition and Food Science, Tabriz University of Medical Sciences, Tabriz, Iran; ^5^ School of Medicine, Semnan University of Medical Sciences, Semnan, Iran; ^6^ Maternal and Childhood Obesity Research Center, Urmia University of Medical Sciences, Urmia, Iran

**Keywords:** interleukin-6, C-reactive protein, antioxidant, biomarkers, coenzyme Q10, umbrella meta-analysis

## Abstract

**Introduction:** Although several meta-analyses support the positive effect of coenzyme Q10 (CoQ10) on biomarkers of oxidative stress and inflammation, the results of some other studies reject such effects.

**Methods:** Therefore, in this umbrella meta-analysis, we performed a comprehensive systematic search in such databases as Web of Science, PubMed, Scopus, Embase, and Google Scholar up to January 2023.

**Results:** Based on standardized mean difference analysis, CoQ10 supplementation significantly decreased serum C-reactive protein (CRP) (ES_SMD_ = −0.39; 95% CI: 0.77, −0.01, *p* = 0.042) and malondialdehyde (MDA) (ES_SMD_ = −1.17; 95% CI: 1.55, −0.79, *p* < 0.001), while it increased the total antioxidant capacity (TAC) (ES_SMD_ = 1.21; 95% CI: 0.61, 1.81, *p* < 0.001) and serum superoxide dismutase (SOD) activity (ES_SMD_ = 1.08; 95% CI: 0.37, 1.79, *p* = 0.003). However, CoQ10 supplementation had no significant reducing effect on tumor-necrosis factor-alpha (TNF- α) (ES_SMD_ = −0.70; 95% CI: 2.09, 0.68, *p* = 0.320) and interleukin-6 (IL-6) levels (ES_SMD_ = −0.85; 95% CI: 1.71, 0.01, *p* = 0.053). Based on weighted mean difference analysis, CoQ10 supplementation considerably decreased TNF-α (ES_WMD_ = −0.46, 95% CI: 0.65, −0.27; *p* < 0.001), IL-6 (ES_WMD_ = −0.92, 95% CI: 1.40, −0.45; *p* < 0.001), and CRP levels (effect sizes _WMD_ = −0.28, 95% CI: 0.47, −0.09; *p* < 0.001).

**Discussion:** The results of our meta-analysis supported the alleviating effects of CoQ10 on markers of inflammation cautiously. However, CoQ10 had antioxidant effects regarding the improvement of all the studied antioxidant and oxidative stress biomarkers.

**Systematic Review Registration:** https://www.crd.york.ac.uk/prospero/display_record.php?RecordID=323861, identifier CRD42022323861

## 1 Introduction

Inflammation is a biological and physiological response of the immune system against infection and tissue injury (Hotamisligil. 2017). However, increased levels of reactive oxygen species (ROS) following chronic inflammation and reduced antioxidant capacity, known as oxidative stress, can cause structural damage to cells (Zhang et al., 2017). A number of transcription factors can be activated by oxidative stress, leading to differential expression of several genes linked to inflammatory pathways. Therefore, oxidative stress is in relation with inflammation, and each can easily induce the other (Bessler et al., 2010; Tarry-Adkins et al., 2016; Mancini et al., 2021). Besides, both inflammation and oxidative stress can be associated with the development of chronic diseases such as diabetes mellitus (DM), obesity, metabolic syndrome, autoimmune diseases, and various types of cancers (Prasad et al., 2016; Ellulu et al., 2017).

Different types of markers are used to detect oxidative and inflammatory stress. Superoxide dismutase (SOD) and glutathione peroxidase (GPx) are the first line of antioxidant defense. SOD metabolizes superoxide radicals and GPx breaks down hydroperoxides into harmless molecules (Zarezadeh et al., 2022). Total antioxidant capacity (TAC) is the measure of the amount of ROS removed by a test solution, being used to assess the antioxidant capacity of biological samples (Rubio et al., 2016). Malondialdehyde (MDA) is used as a marker of free radical formation by lipid peroxidation (Musazadeh et al., 2021). The production of free radicals leads to the translocation of the nuclear-factor-Kappa-B (NF-kB) molecule into the nucleus and the production of pro-inflammatory cytokines such as tumor-necrosis factor-alpha (TNF- α) and interleukin-6 (IL-6).

The scientific community is persistently seeking to find nutrients or compounds that have anti-inflammatory, immunomodulatory, and antioxidant properties, particularly for diseases whose prevention is influenced by unhealthy diet. Coenzyme Q10 (CoQ10) has been discussed as a potential treatment option for chronic diseases in which oxidative stress plays a significant pathophysiological role (Arenas-Jal et al., 2020; Gutierrez-Mariscal et al., 2020; Mancini et al., 2021).

CoQ10 is one of the non-enzymatic antioxidants (Cicero et al., 2018; Samimi et al., 2019), which has both endogenous and exogenous sources (Fakhrolmobasheri et al., 2023). However, its endogenous biosynthesis is impaired in some conditions; therefore, CoQ10 supplementation can be useful in these conditions. In a study, endogenous CoQ10 biosynthesis decreased in patients with type 2 diabetes mellitus (T2DM) compared to subjects with normal glucose tolerance (Gholami et al., 2018). This could be due to impaired CoQ10 metabolism in T2DM or statin therapy in these patients, which may contribute to a decrease in the synthesis of CoQ10 substrates (Gholami et al., 2018). CoQ10 is involved in mitochondrial bioenergetics as well as ROS scavenging due to its participation in redox reactions (Abdeen et al., 2020; Hidalgo-Gutiérrez et al., 2021). Also, CoQ10 can inhibit inflammation through modulation of NF-kB-related pathways (Shukla and Dubey. 2018). Thus, CoQ10 has been proposed as a potential anti-inflammatory and antioxidant agent (Hernández-Camacho et al., 2018).

Several meta-analyses have reported controversial results related to the effect of CoQ10 on oxidative stress and inflammation biomarkers. In a study by Fan et al. (2017), CoQ10 significantly lowered inflammatory factors [TNF-α, IL-6, and C-reactive protein (CRP)], while its supplementation did not affect these factors in other studies (Farsi et al., 2019; Jorat et al., 2019; Dludla et al., 2020). In addition, supplementation with CoQ10 significantly increased TAC and serum SOD activity in several studies (Jorat et al., 2019; Akbari et al., 2020; Sangsefidi et al., 2020; Hajiluian et al., 2021). However, other studies reported that CoQ10 intake could not affect MDA (Dludla et al., 2020) and TAC (Hajiluian et al., 2021). Therefore, the present umbrella meta-analysis aimed to examine the effects of CoQ10 supplementation on serum TNF-α, IL-6, CRP, MDA, SOD, and TAC pooling from the selected meta-analyses. Given that different studies have used various statistical methods and reported diverging results, we used a single statistical method in this study to reach a definite conclusion regarding the anti-inflammatory and antioxidant effects of CoQ10.

## 2 Methods

This study was carried out according to the Preferred Reporting Items for Systematic Reviews and Meta-analysis (PRISMA) guidelines (Moher et al., 2015). Also, we registered the study protocol in PROSPERO (**CRD42022323861**).

### 2.1 Search strategy and study selection

To find the related literature, we systematically searched databases including Web of Science, PubMed, Scopus, Embase, and Google Scholar up to January 2023. The search strategy was developed using the following MeSH terms and keywords (Q10 [Mesh] OR “coenzyme Q10” [tiab] OR ubidecarenone [tiab] OR ubiquinone [Mesh] OR “Bio-Quinone Q10” [tiab] OR ubiquinol [tiab] OR “ubiquinol-10” [tiab]) **AND** (inflammation [Mesh] OR “C-Reactive Protein” [Mesh] OR “c-reactive protein” [tiab] OR crp [tiab] OR “hs-crp” [tiab] OR “high sensitivity-CRP” [tiab] OR “high sensitivity C-reactive protein” [tiab] OR “Tumor Necrosis Factor-alpha” [Mesh] OR “tumor necrosis factor-alpha” [tiab] OR “tumor necrosis factor-α” [tiab] OR “tnf-alpha” [tiab] OR “tnf-α” [tiab] OR “Interleukin-6” [Mesh] OR “interleukin-6” [tiab] OR IL-6 [tiab] OR “interleukin 6” [tiab] OR “Oxidative Stress” [MeSH] OR “Oxidative Stress” [tiab] OR “Total Antioxidant Capacity” [tiab] OR antioxidant [tiab] OR Oxidant [tiab] OR “reactive oxygen species” [tiab] OR Malondialdehyde [tiab] OR glutathione [tiab] OR TAC [tiab] OR GSH [tiab] OR MDA [tiab]) **AND** (“systematic review’’ [Publication Type] OR “meta-analysis” [tiab]). To increase the sensitivity of search strategy, the wild-card term “*’’ was used.

### 2.2 Inclusion and exclusion criteria

We included all meta-analyses of randomized controlled trials (RCTs) evaluating the effects of CoQ10 supplementation on biomarkers of inflammatory and stress oxidative, including TNF-α, IL-6, CRP, MDA, TAC, and SOD in adults (>18 years old). All the included studies reported the estimates of effect sizes (ES) and corresponding confidence intervals (CIs). We also excluded the following studies: *in vitro*, *in vivo*, and *ex vivo* studies; quasi-experimental studies; observational studies; case reports; editorials; and controlled clinical trials. In addition, studies on infants and juveniles were excluded. We also screened the reference lists of all studies manually to ensure the inclusion of all the related literature. Furthermore, we only included articles written in English language.

### 2.3 Study selection and data extraction

Two independent reviewers (ZK and VM) screened the articles according to the eligibility criteria. First, the title and abstract of the retrieved articles were reviewed. Next, we assessed the full-text of all the relevant articles to determine their eligibility for inclusion in the meta-analyses. Any disagreements were resolved by consensus with the third author (AHF).

We extracted the following information from the included meta-analyses: first author’s name, year of publication, sample size, study location, dose and duration range of supplementation, type of ESs [(weighted mean difference (WMD) and standardized mean difference (SMD)], as well as CIs for TNF- α, IL-6, CRP, MDA, TAC, and SOD.

### 2.4 Quality assessment and assessment of the meta-evidence

Methodological quality was assessed independently by two reviewers (MSH and VM) using the AMSTAR2 questionnaire. The AMSTAR2 questionnaire consists of 16 items that ask reviewers to choose one of the following options: “Yes” or “Partial Yes” or “No” or “No Meta-analysis”. The AMSTAR2 checklist was categorized into “critically low quality”, “low quality”, “moderate quality”, and “high quality” (Shea et al., 2017).

The GRADE (Grading of Recommendations, Assessment and Evaluation) approach was used to evaluate the credibility of the included meta-analyses. This approach includes five factors, including risk of bias, consistency of results, directness, precision, and potential for publication bias. The evidence is finally classified into four categories of high, moderate, low, or very low (Guyatt et al., 2008).

### 2.5 Data synthesis and statistical analysis

To estimate overall ES, we used the reported ESs and CIs. Analysis was performed separately for SMD and WMD due to their natural differences. Cochran’s Q test and *I* ^*2*^ statistics were used to determine the heterogeneity. In the current study, *p*-values less than 0.10 and *I* ^*2*^ values higher than 50% were considered as significant for between-study heterogeneity. A random-effects model was performed using the restricted maximum likelihood (REML) strategy when between-study heterogeneity was critical (*I* ^*2*^ > 50% or *p* < 0.1). To explore the sources of heterogeneity, we performed subgroup analysis by duration, mean age, sample size, and dose. The sensitivity analysis was conducted to determine whether removing a single study would affect the overall ES. Publication bias was assessed using funnel plots, and the Egger’s test was applied if the number of included datasets was ten or higher; otherwise, only the Begg’s test result was reported. To simulate a model without publication bias, the trim-and-fill method was used when publication bias was detected. All statistical analyses were carried out using STATA, version 16 (Stata Corporation, College Station, TX, US). *p*-values less than 0.05 were considered significant.

## 3 Results

### 3.1 Systematic review

**FIGURE 1:**
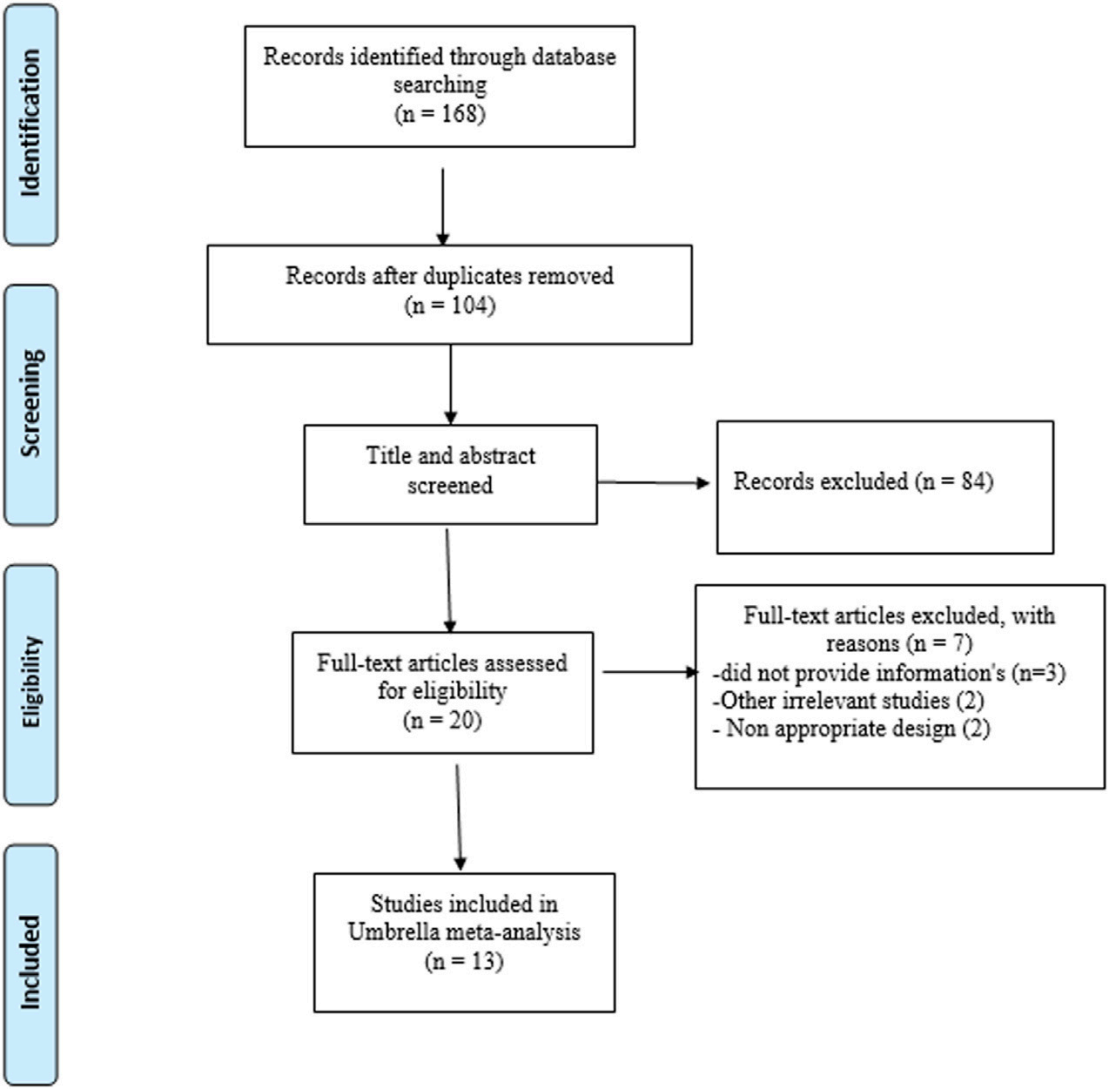
PRISMA flow diagram of selection studies.

The flowchart of the search process is presented in Figure 1. After screening all the related data, a total of 13 meta-analyses published between 2017 and 2022 were included in our umbrella meta-analysis. The characteristics of the included studies are summarized in Table 1. The participants’ ages ranged from 43 to 69 years. Based on the country of the first author, the studies were as follows: seven in Iran (Farsi et al., 2019; Jorat et al., 2019; Akbari et al., 2020; Sangsefidi et al., 2020; Alimohammadi et al., 2021; Hajiluian et al., 2021; Sedaghat et al., 2022), five in China (Fan et al., 2017; Zhai et al., 2017; Mazidi et al., 2018; Zhang et al., 2019; Dai et al., 2022), and one in South Africa (Dludla et al., 2020). Interventions varied in duration, from 6 to 20 weeks, and supplement doses varied from 65 to 300 mg/day. There were also differences in the health status of the participants (Table 1). The quality of the trials was assessed using the “Jadad score” (Clark et al., 1999) system and the Cochrane risk of bias tool (Higgins et al., 2011). Overall, nearly 90% of the included meta-analyses contained high-quality RCTs (Table 1).

**TABLE 1:** Characteristics of the included studies.

Citation (first author et al., year)	Location	Study population	Sample size	Mean age	Q_10_ dosage (mg)	Duration (Week)	Main outcome
Zhai et al. (2017)	China	NAFLD, MS, CAD, MI	180	46.9	275	12	IL-6 ↔
		risk of CVD in CKD, obesity, CAD, NAFLD, MetS, HTN	331	52.2	185.7	10	CRP ↔
		NAFLD, MS, CAD	128	43.5	300	12	TNF-α↓
Fan et al. (2017)	China	runners, NAFLD, CAD, mild hypertensive, RA, renal lithiasis, hyperlipidemic with MI, MS	402	45.5	201.1	9	IL-6↓
		runners, NAFLD, CAD, end stage heart failure, RA, MS	217	43.6	194.8	9.5	TNF-α ↓
		systolic dysfunction, runners, T2DM, NAFLD, obese, CAD, hypercholesterolemia, chronic renal impairment, mild hypertensive, heart failure, ESRD	543	53.4	159	11	CRP ↓
Mazidi et al. (2018)	China	ischemic LVSD, DM with neuropathy, BMI>25, CAD	385	65.9	188.75	11	IL-6↓
		stenosis of one major, coronary artery, chronic renal impairment, mildly hypertensive					CRP↔
Jorat et al. (2019)	Iran	CAD, AMI, MI	295	54.4	114	13	MDA↓
		CHF, end stage heart failure, CAD	102	66	170	20	TNF-α ↔
		CAD, T2DM with coronary heart disease	155	68.3	152.5	11	IL-6 ↔
		CAD, systolic dysfunction, CHF, ischemic heart disease	313	63.6	218.3	10.5	CRP↔
		CAD, ischemic heart disease, systolic dysfunction	184	67.9	222	10	SOD↑
Zhang et al. (2019)	China	CKD	115	67.2	65	12	MDA↓
Farsi et al. (2019)	Iran	migraine, HCC, NAFLD, RA, mild HTN, MS, CAD, end-stage heart failure	348	48.7	232.5	11.5	TNF-α ↔
		migraine, HCC, T2DM, NAFLD, RA, mild HTN, MS, HLP with MI, CAD	454	55.9	210	11	IL-6 ↓
		HCC, NAFLD, mild HTN, CAD	208	65.4	168.3	12	CRP ↔
Dludla et al. (2020)	South Africa	mildly hypertensive, NAFLD	101	NR	100	12	TNF-α↔
							CRP ↓
		NAFLD, T2DM	161	47.5	366.6	6.5	MDA↔
Akbari et al. (2020)	Iran	HD, T2DM, T1DM, bipolar disorders, acute coronary syndrome, RA, NAFLD	540	48.8	114	11.5	MDA ↓
		bipolar disorders, HD, T2DM, RA, NAFLD	464	52.8	165	11	TAC↑
		Hepatocellular carcinoma, renal injury, CAD, systolic dysfunction	237	62.2	275	8	SOD↑
Sangsefidi et al. (2020)	Iran	T2D, ESRF, healthy, CRF, DN, HCC, MS, CAD, RA, NAFLD	715	NR	182.1	11	MDA↓
		NAFLD, RA, MS, dyslipidemia, T2DM	481	NR	167.4	12	TAC↑
		ischemic LVSD, CAD, MS, HCC, T2DM	284	NR	244.2	11.5	SOD↑
Hajiluian et al. (2021)	Iran	RA, kidney disease, NAFLD, T2DM, CVD	480	53.9	104.4	11	MDA ↓
		T2DM	236	57.2	100	10	MDA ↓
		RA, kidney disease, NAFLD, CVD	244	50.6	108	12	MDA↓
		RA, T2DM, kidney disease, NAFLD	406	54.8	160	11.5	TAC↑
		RA, NAFLD, kidney disease	148	51.3	106.6	8	TAC↔
		T2DM	258	57.5	200	14	TAC↑
		kidney disease, CVD, cancer	248	62.2	275	8.5	SOD↑
Alimohammadi et al. (2021)	Iran	breast cancer	156	57	100	9.5	SOD↑
							TNF-α↓
							IL-6↓
Dai et al. (2022)	China	Healthy, dyslipidemia, NAFLD, T2DM, hepatocellular carcinoma, coronary artery disease, bipolar disorder, MS, hemodialysis	1912	50	200	11.5	MDA ↓
							TAC↑
							SOD↔
Sedaghat et al. (2022)	Iran	Healthy, dyslipidemia, NAFLD, T2DM, hepatocellular carcinoma, coronary artery disease, bipolar disorder, MS, renal injury, ICU patients, autism, migraine	1,267	NR	195	9	MDA ↓
							TAC↑
							SOD↑

Abbreviations: NR, not reported; NAFLD, nonalcoholic fatty liver disease; MS, multiple sclerosis; CAD, coronary artery disease; MI, myocardial infarction; MetS, metabolic syndrome; HTN, hypertension; RA, rheumatoid arthritis; T2DM, Type 2 diabetes; CVD, cardiovascular disease; ESRD, End-Stage Renal Disease; LVSD, Left Ventricular Systolic Dysfunction; HCC, hepatocellular carcinoma; DN, diabetic nephropathy; AMI, acute myocardial infarction.

### 3.2 Risk of bias assessment and quality of evidence

Applying the AMSTAR 2 tool showed that the meta-analyses are of high quality (Table 2). Out of 13 meta-analyses, ten studies had high quality and three studies had moderate quality. Regarding SMD and WMD analyses, while biomarkers had high-quality evidence, oxidative stress had low-quality evidence (Table 3).

**TABLE 2:** Results of assessing the methodological quality of all the meta-analyses included in the meta-analysis.

Study	Q1^1^	Q2	Q3	Q4	Q5	Q6	Q7	Q8	Q9	Q10	Q11	Q12	Q13	Q14	Q15	Q16	Quality assessment
Zhai et al. (2017)	No	Partial Yes	Yes	Partial Yes	Yes	Yes	Partial Yes	Yes	Yes	No	Yes	Yes	Yes	Yes	Yes	No	Moderate
Fan et al. (2017)	No	Partial Yes	Yes	Yes	Yes	Yes	Yes	Yes	Yes	Yes	Yes	Yes	Yes	Yes	Yes	Yes	High
Mazidi et al. (2018)	No	Yes	Yes	Yes	Yes	Yes	Yes	Yes	Yes	No	Yes	Yes	Yes	Yes	Yes	Yes	High
Hajiluian et al. (2021)	No	Yes	Yes	Partial Yes	Yes	Yes	Yes	Yes	Yes	Yes	Yes	Yes	Yes	Yes	Yes	Yes	High
Jorat et al. (2019)	No	Partial Yes	Yes	Partial Yes	Yes	Yes	Yes	Yes	Yes	Yes	Yes	Yes	Yes	Yes	Yes	Yes	High
Zhang et al. (2019)	No	Partial Yes	Yes	Partial Yes	Yes	Yes	Yes	Yes	Yes	No	Yes	Yes	Yes	Yes	Yes	No	Moderate
Farsi et al. (2019)	No	Partial Yes	Yes	Partial Yes	No	Yes	No	Yes	Yes	No	Yes	No	Yes	Yes	Yes	Yes	Moderate
Dludla et al. (2020)	No	Yes	Yes	Partial Yes	Yes	Yes	Yes	Yes	Yes	Yes	Yes	Yes	Yes	No	Yes	Yes	High
Alimohammadi et al. (2021)	No	Yes	Yes	Partial Yes	Yes	Yes	Yes	Yes	Yes	No	Yes	Yes	Yes	Yes	Yes	Yes	High
Akbari et al. (2020)	No	Yes	Yes	Partial Yes	Yes	Yes	Yes	Yes	Yes	No	Yes	Yes	Yes	Yes	Yes	Yes	High
Sangsefidi et al. (2020)	No	Partial Yes	Yes	Yes	Yes	Yes	Yes	Yes	Yes	No	Yes	Yes	Yes	Yes	Yes	Yes	High
Dai et al. (2022)	No	Yes	Yes	Partial Yes	Yes	Yes	Yes	Yes	Yes	No	Yes	Yes	Yes	Yes	Yes	Yes	High
Sedaghat et al. (2022)	No	Partial Yes	Yes	Partial Yes	Yes	Yes	Yes	Yes	Yes	No	Yes	Yes	Yes	Yes	Yes	Yes	High

Did the research questions and inclusion criteria for the review include the components of PICO? 2. Did the report of the review contain an explicit statement that the review methods were established prior to the conduct of the review and did the report justify any significant deviations from the protocol? 3. Did the review authors explain their selection of the study designs for inclusion in the review? 4. Did the review authors use a comprehensive literature search strategy? 5. Did the review authors perform study selection in duplicate? 6. Did the review authors perform data extraction in duplicate? 7. Did the review authors provide a list of excluded studies and justify the exclusions? 8. Did the review authors describe the included studies in adequate detail? 9. Did the review authors use a satisfactory technique for assessing the risk of bias (RoB) in individual studies that were included in the review? 10. Did the review authors report on the sources of funding for the studies included in the review? 11. If meta-analysis was performed, did the review authors use appropriate methods for statistical combination of results? 12. If meta-analysis was performed, did the review authors assess the potential impact of RoB in individual studies on the results of the meta-analysis or other evidence synthesis? 13. Did the review authors account for RoB in individual studies when interpreting/discussing the results of the review? 14. Did the review authors provide a satisfactory explanation for, and discussion of, any heterogeneity observed in the results of the review? 15. If they performed quantitative synthesis, did the review authors carry out an adequate investigation of publication bias (small study bias) and discuss its likely impact on the results of the review? 16. Did the review authors report any potential sources of conflict of interest, including any funding they received for conducting the review?

Each question was answered with “Yes”, “Partial Yes” or “No”. When no meta-analysis was done, question 11, 12 and 15 were answered with “No meta-analysis conducted.

**TABLE 3:** Summary of findings and quality of evidence of the CoQ10 supplementation on inflammatory biomarkers.

Outcome measures	Summary of findings		Quality of evidence assessment (GRADE)					
	No of patients (meta-analysis)	Effect size (95% CI)	Risk of bias^a^	Inconsistency^b^	Indirectness^c^	Imprecision^d^	Publication bias^e^	Quality of evidence^f^
**SMD analysis**								
CRP	622 (3)	−0.39 (−0.77, −0.01)	Not Serious	Not Serious	Serious	Not Serious	Not Serious	High
TNF-α	885 (4)	−0.70 (−2.09, 0.68)	Not Serious	Not Serious	Serious	Not Serious	Not Serious	High
IL-6	1,076 (4)	−0.85 (−1.71, 0.01)	Not Serious	Not Serious	Serious	Not Serious	Not Serious	High
MDA	4,550 (9)	−1.17 (−1.55, −0.79)	Not Serious	Serious	Serious	Serious	Not Serious	Low
TAC	2,796 (6)	1.21 (0.61, 1.81)	Not Serious	Serious	Serious	Serious	Not Serious	Low
SOD	2,280 (7)	1.08 (0.37, 1.79)	Not Serious	Serious	Serious	Serious	Not Serious	Low
**WMD analysis**								
CRP	1,259 (3)	−0.28 (-0.47, −0.09)	Not Serious	Not Serious	Serious	Not Serious	Not Serious	High
TNF-α	345 (2)	−0.46 (−0.65, −0.27)	Not Serious	Not Serious	Serious	Not Serious	Not Serious	High
IL-6	967 (3)	−0.92 (−1.40, −0.45)	Not Serious	Not Serious	Serious	Not Serious	Not Serious	High

CRP, C-reactive protein; TNF, tumor necrosis factor; IL-6, interleukin-6; TAC, total antioxidant capacity; MDA, malondialdehyde; SOD, superoxide dismutase.

^a^ Risk of bias according to AMSTAR2 results.

^b^ Downgraded if there was a substantial unexplained heterogeneity (*I* ^2^ > 50%, *p* < 0.10) that was unexplained by meta-regression or subgroup analyses.

^c^ Downgraded if there were factors present relating to the participants, interventions, or outcomes that limited the generalizability of the results. Participants of the included studies were from different health conditions (subgroup analysis was not performed for each disease).

^d^ Downgraded if the 95% confidence interval (95% CI) crossed the minimally important difference (MID) for benefit or harm. MIDs, used for each outcome were: 3.16 mg/L for CRP, 7.9 pg/mL for TNF-α, and 2 pg/mL for IL-6, 0.59 mmol/mL for MDA, and 0.08 mmol/L for TAC.

^e^ Downgraded if there was an evidence of publication bias using funnel plot.

^f^ Since all the included studies were meta-analyses, the certainty of the evidence was graded as high for all outcomes by default and then downgraded based on prespecified criteria. Quality was graded as high, moderate, low, or very low.

### 3.3 Effects of CoQ10 supplementation on TNF-α based on SMD analysis

**FIGURE 2:**
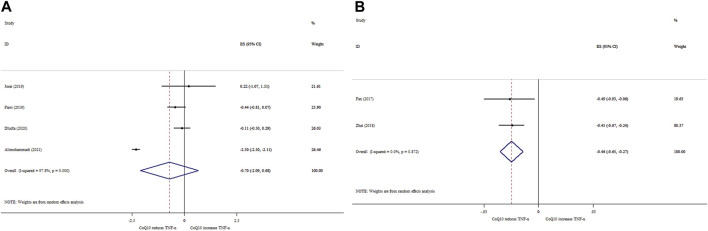
Forest plot of impacts of CoQ10 supplementation on TNF-α based on SMD **(A)** and WMD **(B)** analysis.

Data from four meta-analyses indicated that CoQ10 supplementation did not significantly reduce TNF-α levels (ES_SMD_ = −0.70; 95% CI: 2.09, 0.68, *p* = 0.320) (Figure 2A). Meanwhile, between-study heterogeneity was found to be quite high (*I* ^*2*^ = 97.8%, *p* < 0.001). In contrast to overall effect, subgroup analysis showed that CoQ10 supplementation significantly decreased TNF-α levels when intervention duration and dose were ≤10 weeks and >200 mg/day, respectively (Table 4). According to the sensitivity analysis, excluding any of the studies did not affect the estimate of the overall ES. No indication of publication bias was observed according to the Begg’s test (*p* = 0.707).

**TABLE 4:** Pooled estimates of the effect of coenzyme Q10 on inflammatory and stress oxidative biomarkers according to SMD analysis.

Group	No. of comparisons	SMD (95% CI)	*p*-value	*I* ^*2*^ (%)	P-heterogeneity
**Q10 supplementation on TNF-α levels**					
**Total**	4	−0.70 (−2.09, 0.68)	0.320	97.8	<0.001
**Age (years)**					
≤55	1	−0.44 (−0.88, - 0.00)	0.050	-	-
>55	2	−1.12 (−3.59, 1.34)	0.371	93.0	<0.001
NR	1	−0.11 (−0.50, 0.28)	0.585	-	-
**Duration (week)**					
≤10	1	−2.30 (−2.49, −2.11)	<0.001	-	-
>10	3	−0.23 (−0.52, 0.05)	0.110	0.0	0.428
**Dose (mg)**					
≤200	3	−0.78 (−2.58, 1.01)	0.392	98.1	<0.001
>200	1	−0.44 (−0.88, −0.00)	0.050	-	-
**Q10 supplementation on IL-6 levels**					
**Total**	4	−0.85 (−1.71, 0.01)	0.053	95.9	<0.001
**Age(years)**					
≤55	1	−1.56 (−1.73, −1.39)	<0.001	-	-
>55	2	−0.67 (−1.73, 0.38)	0.211	45.6	0.175
NR	1	−0.24 (−0.63, 0.16)	0.234	-	-
**Duration (weeks)**					
≤10	1	−1.56 (−1.73, −1.39)	<0.001	-	-
>10	3	−0.35 (−0.61, −0.09)	0.008	11.5	0.323
**Dose (mg)**					
≤200	3	−1.06 (−2.17, 0.06)	0.064	94.5	<0.001
>200	1	−0.37 (−0.65, −0.09)	0.010	-	-
**Q10 supplementation on CRP levels**					
**Total**	3	−0.39 (−0.77, −0.01)	0.042	38.5	0.197
**Q10 supplementation on MDA levels**					
**Total**	9	−1.17 (−1.55, −0.79)	<0.001	62.4	0.007
**Sample size**					
≤300	5	−1.36 (−2.17, −0.56)	<0.001	60.5	0.038
>300	4	−1.15 (−1.65, −0.66)	<0.001	73.0	0.011
**Age (year)**					
≤55	5	−1.13 (−1.69, −0.57)	<0.001	56.4	0.057
>55	2	−0.79 (−1.28, −0.31)	<0.001	16.2	0.275
NR	2	−1.84 (−3.42, −0.25)	0.023	84.8	0.010
**Duration (weeks)**					
≤10	3	−1.60 (−3.11, −0.09)	0.038	82.3	0.004
>10	6	−1.07 (−1.45, −0.70)	<0.001	49.8	0.076
**Dose (mg/day)**					
≤200	8	−1.16 (−1.55, −0.77)	<0.001	66.5	0.004
>200	1	−1.57 (−3.61, 047)	0.131	-	-
**Q10 supplementation on TAC levels**					
**Total**	6	1.21 (0.61, 1.81)	<0.001	76.9	<0.001
**Sample size**					
≤300	2	0.66 (0.16, 1.16)	0.010	19.4	0.265
>300	4	1.65 (0.67, 2.62)	<0.001	83.6	<0.001
**Age (year)**					
≤55	3	0.92 (0.13, 1.70)	0.022	77.7	0.011
>55	1	0.84 (0.32, 1.36)	0.002	-	-
NR	2	2.27 (0.21, 4.34)	0.031	82.9	0.016
**Duration (weeks)**					
≤10	2	1.79 (−1.24, 4.83)	0.247	92.8	<0.001
>10	4	1.07 (0.58, 1.56)	<0.001	61.2	0.052
**Dose (mg/day)**					
≤200	4	0.73 (0.45, 1.01)	<0.001	0.0	0.435
>200	2	2.49 (0.97, 4.01)	<0.001	72.5	0.057
**Q10 supplementation on SOD levels**					
**Total**	7	1.08 (0.37, 1.79)	0.003	95.7	<0.001
**Sample size**					
≤200	2	2.43 (2.14, 2.72)	<0.001	0.0	0.782
>200	5	0.48 (0.32, 0.64)	<0.001	0.0	0.420
**Age (year)**					
≤60	2	1.45 (−0.46, 3.36)	0.136	97.9	<0.001
>60	3	0.62 (0.11, 1.12)	0.017	77.3	0.012
NR	2	0.77 (0.26, 1.28)	0.003	33.1	0.222
**Duration (weeks)**					
≤10	5	0.98 (0.06, 1.89)	0.036	96.9	<0.001
>10	2	1.49 (−0.46, 3.45)	0.134	85.5	0.009
**Dose (mg/day)**					
≤200	1	2.42 (2.13, 2.72)	<0.001	-	-
>200	6	0.59 (0.32, 0.87)	<0.001	58.8	0.033

**Abbreviation:** N, number; NR, not reported.

### 3.4 Effects of CoQ10 supplementation on TNF-α based on WMD analysis

Our findings based on two meta-analyses revealed that CoQ10 supplementation considerably decreased TNF-α levels (ES_WMD_ = −0.46, 95% CI: 0.65, −0.27; *p* < 0.001), with no considerable between-study heterogeneity (*I* ^*2*^ = 0.0%; *p* = 0.872) (Figure 2B).

### 3.5 Effects of CoQ10 supplementation on IL-6 based on SMD analysis

**FIGURE 3:**
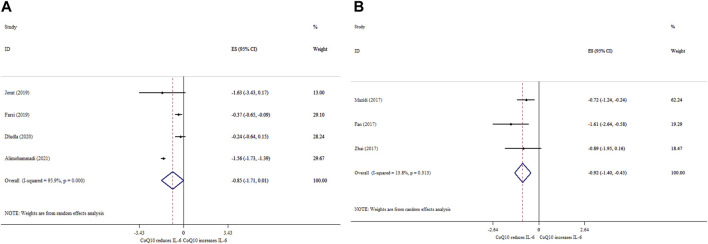
Forest plot of impacts of CoQ10 supplementation on IL-6 based on SMD **(A)** and WMD **(B)** analysis.

CoQ10 supplementation did not significantly reduce IL-6 levels (ES_SMD_ = −0.85; 95% CI: 1.71, 0.01, *p* = 0.053) (Figure 3A). Also, a high degree of heterogeneity was detected (*I* ^*2*^ = 95.9%, *p* < 0.001). Subgroup analysis revealed that the ameliorative effects of CoQ10 supplementation on the IL-6 levels were stronger when the treatment dose was >200 mg/day, the duration was ≤10 weeks, and age was ≤55 years (Table 4). Furthermore, the overall effects of CoQ10 on IL-6 changed to be statistically significant when the studies were removed using sensitivity analysis (Dludla et al., 2020; Alimohammadi et al., 2021). In addition, the Begg’s test did not reveal any evidence of publication bias (Begg’s, *p* = 0.548).

### 3.6 Effects of CoQ10 supplementation on IL-6 based on WMD analysis

CoQ10 supplementation significantly decreased IL-6 levels (ES_WMD_ = −0.92; 95% CI: 1.40, −0.45, *p* < 0.001; *I* ^*2*^ = 13.8%, *p* = 0.313) (Figure 3B).

### 3.7 Effects of CoQ10 supplementation on CRP based on SMD analysis

**FIGURE 4:**
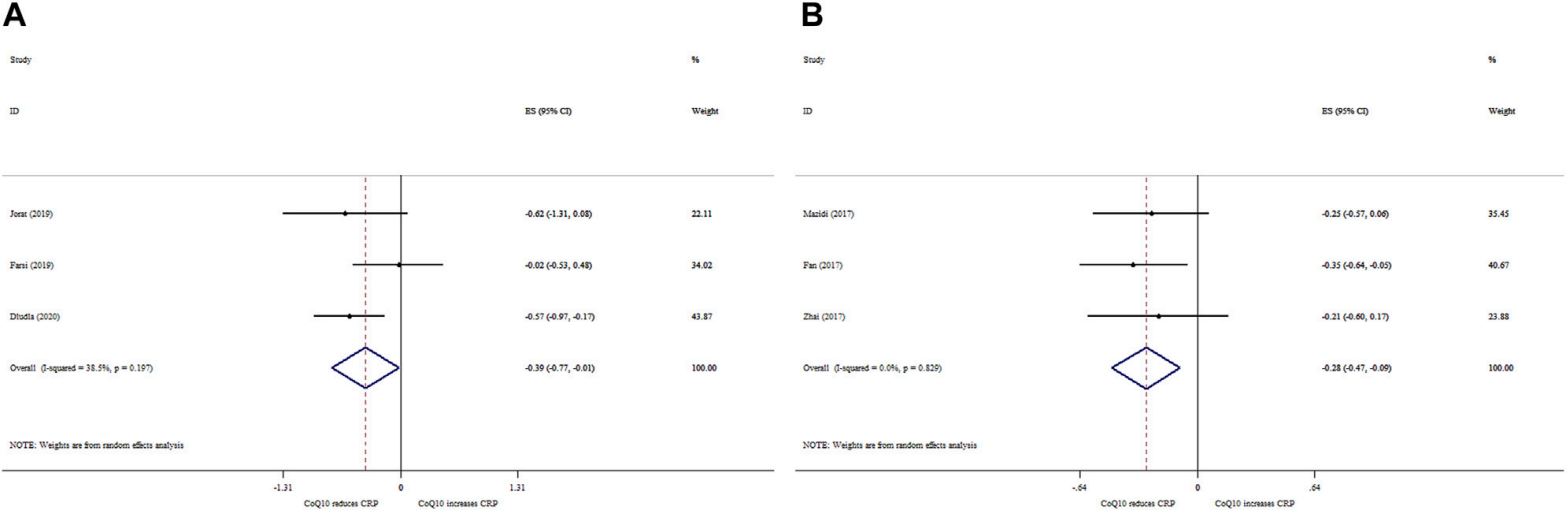
Forest plot of impacts of CoQ10 supplementation on CRP based on SMD **(A)** and WMD **(B)** analysis.

CoQ10 supplementation significantly reduced CRP levels (ES_SMD_ = −0.39; 95% CI: 0.77, −0.01, *p* = 0.042) (Figure 4A). Nevertheless, there was no significant between-study heterogeneity (*I* ^*2*^ = 38.5%, *p* = 0.197).

### 3.8 Effects of CoQ10 supplementation on CRP based on WMD analysis

Pooling three meta-analyses revealed a significant reduction in CRP levels (ES _WMD_ = −0.28, 95% CI: 0.47, −0.09; *p* < 0.001) (Figure 4B), with no considerable between-study heterogeneity (*I* ^*2*^ = 0.0%; *p* = 0.829).

### 3.9 Effects of CoQ10 supplementation on MDA based on SMD analysis

**FIGURE 5:**
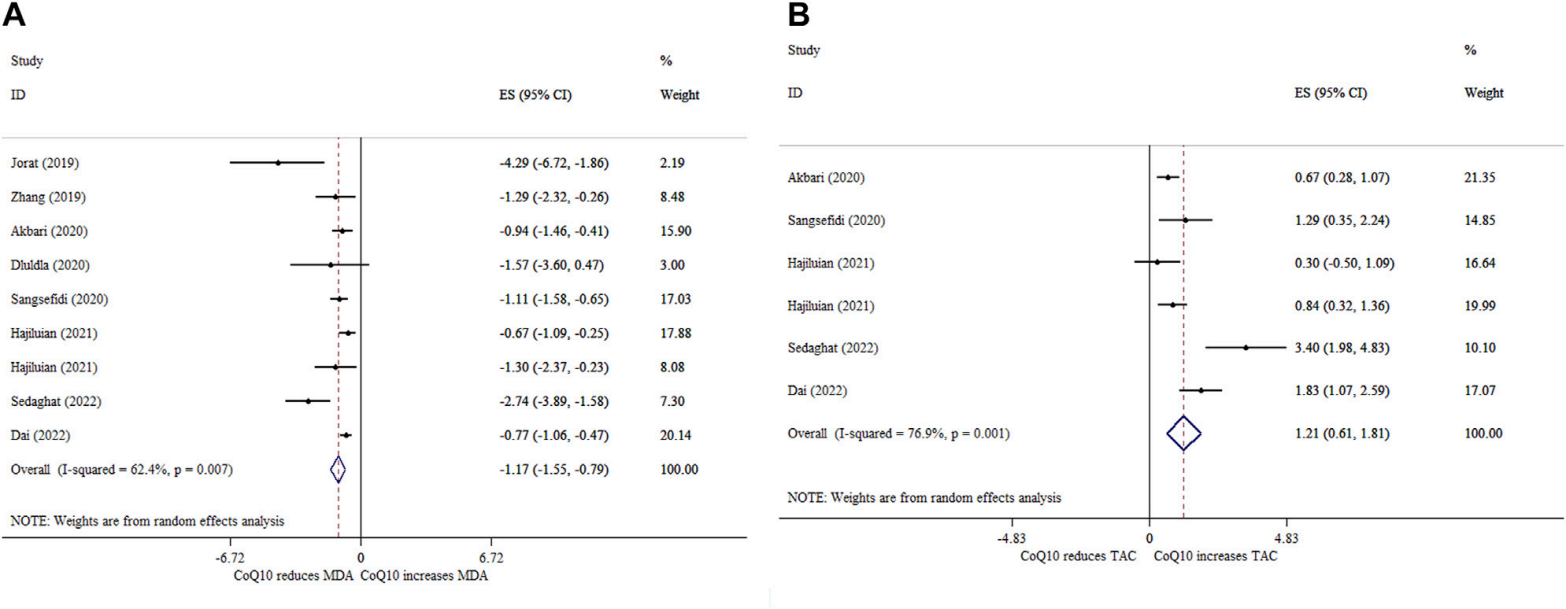
Forest plot of impacts of CoQ10 supplementation on MDA **(A)** and TAC **(B)** levels based on SMD analysis.

Eight meta-analyses with nine ESs reported that CoQ10 supplementation significantly reduced MDA levels (ES_SMD_ = −1.17; 95% CI: 1.55, −0.79, *p* < 0.001) (Figure 5A). Moreover, there was a significant between-study heterogeneity (*I* ^*2*^ = 62.4%, *p* = 0.007) (Table 4). Subgroup analysis demonstrated that CoQ10 supplementation had a greater reducing effect on MDA in people aged under 55 years, intervention duration ≤10 weeks, and sample size ≤300 (Table 4). Sensitivity analysis revealed no change in the direction of ESs when we removed any of the ESs in the overall analysis. No proof of publication bias was observed after performing the Begg’s test (*p* = 0.174).

### 3.10 Effects of CoQ10 supplementation on TAC based on SMD analysis

Five meta-analyses with six ESs indicated that CoQ10 supplementation significantly increased TAC (ES_SMD_ = 1.21; 95% CI: 0.61, 1.81, *p* < 0.001) (Figure 5B). Also, there was a high between-study heterogeneity (*I* ^*2*^ = 76.9%, *p* = 0.435) (Table 4). The effects of CoQ10 on TAC levels at doses >200 mg/day in studies with an intervention duration of >10 weeks and a sample size of >300 participants were stronger than other subgroups (Table 4). According to sensitivity analysis, the overall ES did not change significantly by excluding any of the studies. No evidence of publication bias was found following Begg’s test (*p* = 0.806).

### 3.11 Effects of CoQ10 supplementation on SOD activity *based on SMD analysis*

**FIGURE 6:**
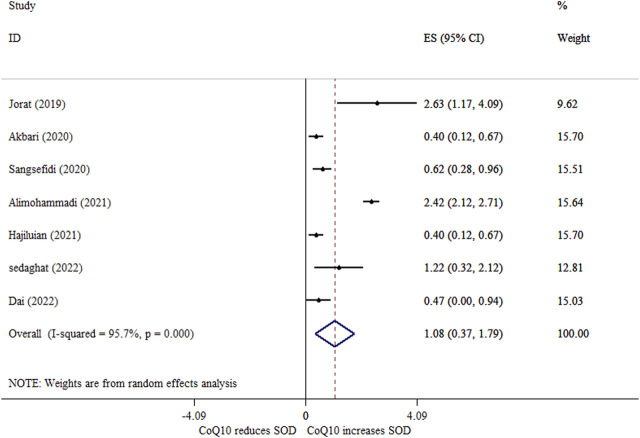
Forest plot of impacts of CoQ10 supplementation on SOD levels based on SMD analysis.

Seven meta-analyses indicated that CoQ10 supplementation significantly increased serum SOD activity (ES_SMD_ = 1.08; 95% CI: 0.37, 1.79, *p* = 0.003) (Figure 6). Moreover, we found a high degree of between-study heterogeneity (*I* ^*2*^ = 95.7%, *p* < 0.001) with sample size as its source (Table 4). Based on the sensitivity analysis results, the overall ES did not depend on a single study. There was no sign of publication bias based on Begg’s test (*p = 0.327*).

## 4 Discussion

The current umbrella meta-analysis evaluated the therapeutic effects of CoQ10 on inflammation and oxidative stress. To this end, we included 13 meta-analyses containing 77 trials. Based on SMD analysis, our findings showed that CoQ10 supplementation enhanced serum activity of SOD and TAC, but it declined CRP and MDA levels. Meanwhile, CoQ10 supplementation did not affect TNF-α and IL-6 levels. However, according to the results of WMD analysis, CoQ10 reduced IL-6 and TNF-α levels. Since the WMD depends only on the weight of each study, it can be concluded that the standard deviation of the ESs related to IL-6 and TNF-α was effective in the final result (Andrade. 2020). Moreover, the limited number of analyzed studies with WMD could reduce the power of its related findings. On the other hand, the high heterogeneity of the analyzed studies with SMD can also question the validity of its related findings. Consequently, definitive conclusions regarding the effect of CoQ10 supplementation on IL-6 and TNF-α should be made with caution.

Due to the limited number of studies that reported WMD, subgroup analysis was performed only for studies that reported SMD. However, the small number of studies in some subgroups led to a low-powered ES. Nonetheless, this limited subgroup analysis also showed that the effect of CoQ10 on inflammatory and oxidative stress indices was not dependent on sample size. Regarding other subgroups, >200 mg/day for ≤10 weeks of CoQ10 supplementation showed more improving outcomes in patients with mean age of ≤55 years. However, there were some exceptions. For example, >10 weeks of CoQ10 supplementation and doses of ≤200 mg/day resulted in further increases in TAC and serum SOD activity, respectively. There are various oral doses for CoQ10 over the counter from 30 mg to 600 mg (Raizner. 2019). However, doses up to 1,200 mg/day have been reported to be tolerated (Garrido-Maraver et al., 2014).

**FIGURE 7:**
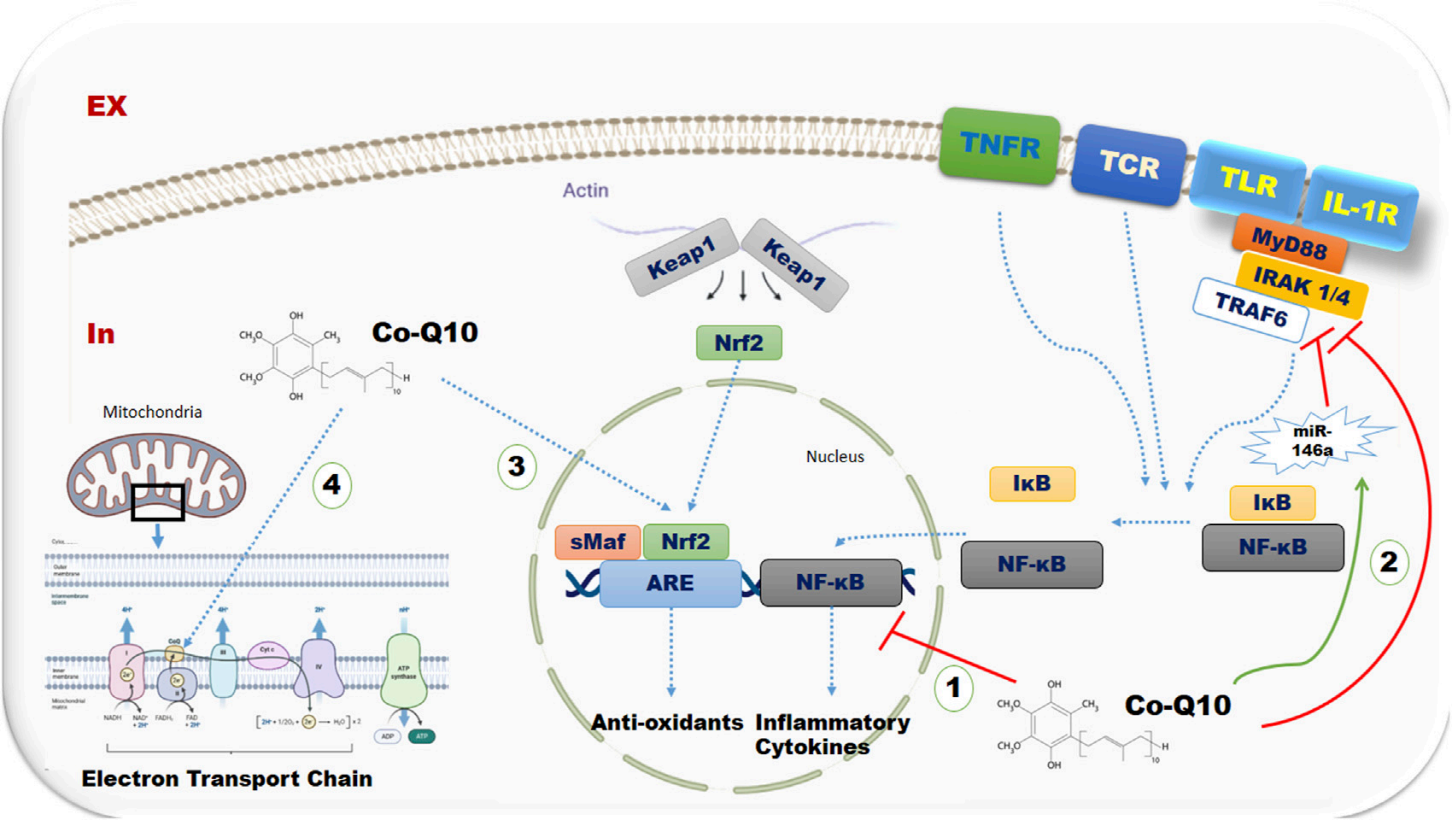
The possible mechanisms of CoQ10 supplementation on inflammatory and oxidative stress biomarkers Abbreviations: TNFR, TNF receptor; ,TLRs, Toll-like receptors; TCR, T lymphocyte receptor; Keap 1, kelch-like ECH-associated protein 1; Nrf2, nuclear factor (erythroid-derived 2)-like 2; NF-kB, nuclear-factor-Kappa-B; IKB, I-kappa-B; miR-146a, microRNAs-146a; MYD88, Myeloid differentiation primary response 88; IRAK4, Interleukin-1 receptor–associated kinase 4; TRAF6, TNF receptor associated factor 6

CoQ10, a well-known nutritional supplement with antioxidant properties, exerts protective roles in various metabolic and inflammatory processes (Zhai et al., 2017). Numerous potential mechanisms can explain these features (Figure 7). CoQ10 may play a role in declining the production of pro-inflammatory cytokines by inhibiting NF-κB gene expression, which is involved in the expression of pro-inflammatory cytokines, such as TNF-α and IL-6 (Schmelzer et al., 2008; Fan et al., 2017). Moreover, CoQ10 modulates the expression of microRNAs-146a (miR-146a), which is an NF-κB-dependent gene. However, through direct downregulation of IL-1 receptor-associated kinase 1 (IRAK-1) and TNF receptor-associated factor 6 (TRAF6), it exerts a negative feedback effect on Toll-like receptor and cytokine signaling, leading to the suppression of NF-κB-mediated inflammatory molecules (Taganov et al., 2006; Schmelzer et al., 2009; Xie et al., 2018; Long et al., 2019). CoQ10 has been reported to have a downregulating effect on the secretion of CRP (Schmelzer et al., 2008) and pro-inflammatory chemokines such as macrophage inflammatory protein-1 alpha (Schmelzer et al., 2007), possibly due to the inhibitory effect of CoQ10 on NF-kB. Moreover, CoQ10 has been found to have an increasing effect on nuclear factor (erythroid-derived 2)-like 2 (Nrf2) and heme oxygenase 1 (HO-1) in the oxidative state (Pala et al., 2016). Nrf2 is a key transcription factor that targets the genes of antioxidant proteins (Gao et al., 2022). Also, it has been suggested that CoQ10 is a peroxisome proliferator-activated receptor gamma and alpha (PPAR-γ and α) ligand (Tiefenbach et al., 2018). Activated PPARs modulate the inflammatory responses through their regulatory effects on the expression of several genes involved in inflammation (Wu et al., 2020). In addition, CoQ10, as a redox carrier in the mitochondrial membrane, declines cellular oxidative stress and free radical production (Sangsefidi et al., 2020).

Although most studies reported beneficial effects of CoQ10 on inflammation and oxidative stress, some other studies showed different findings. The possible reasons for such inconsistencies include differences in study design (Farsi et al., 2019; Alimohammadi et al., 2021), sample size (Zhai et al., 2017; Mazidi et al., 2018; Farsi et al., 2019; Alimohammadi et al., 2021), doses of CoQ10 (Fan et al., 2017; Zhai et al., 2017; Farsi et al., 2019; Jorat et al., 2019; Zhang et al., 2019; Akbari et al., 2020), duration of treatment (Fan et al., 2017; Jorat et al., 2019; Zhang et al., 2019; Akbari et al., 2020; Dludla et al., 2020; Hajiluian et al., 2021), formulation type of the supplement (Fan et al., 2017; Farsi et al., 2019; Sangsefidi et al., 2020), baseline characteristics such as gender, age (Farsi et al., 2019; Zhang et al., 2019; Hajiluian et al., 2021), body mass index (BMI), and lipoprotein concentration (Zhai et al., 2017), as well as low-grade inflammation and/or oxidative stress among healthy subjects (Mazidi et al., 2018; Sangsefidi et al., 2020).

The results of WMD analysis reflected a significant decline in IL-6 and TNF-α levels. Regarding CRP, a significant reduction was observed for both SMD and WMD analyses. In inflammatory pathways, CRP is in the downstream of pro-inflammatory cytokines such as IL-6 and TNF-α, and its hepatic biosynthesis is mainly dependent on IL-6 (Farsi et al., 2019). Accordingly, CRP is an independent risk factor for cardiovascular diseases and diabetes; it also appears to be less sensitive than IL-6 in response to the inflammatory state (Fan et al., 2017; Farsi et al., 2019). The contradictory reports regarding the effect of CoQ10 supplementation on inflammatory markers such as CRP and IL-6 might be attributed to the following two reasons: 1) some studies included healthy subjects with low baseline levels of inflammation that may not be affected by CoQ10, and 2) the dose of CoQ10 supplements may have been too low to observe anti-inflammatory effects. Zhai et al. (2017) mentioned that the association between CRP, IL-6, and CoQ10 serum concentration was influenced by age, sex, BMI, lipoprotein concentration, and health status. Accordingly, many studies have repeatedly shown that individuals with chronic inflammation may be more likely to benefit from CoQ10 intervention (Fan et al., 2017). In fact, differences in baseline levels of oxidative stress indices determine the effectiveness of CoQ10 supplementation on inflammation and oxidative stress (Sangsefidi et al., 2020).

Based on subgroup analyses, CoQ10 supplementation was more effective in reducing inflammation and oxidative stress in subjects younger than 55 years old. Aging increases the production of inflammatory mediators and can continuously diminish the rate of CoQ10 biosynthesis. This supports the hypothesis that exogenous supplementation can compensate for the low levels of CoQ10 (Farsi et al., 2019). As a result, it seems that elderly people should consume more than young people to compensate for this deficiency. Since the mean age of participants in the included studies was 43–69 years old, those under 55 years of age benefited the most from CoQ10 supplementation. However, due to the limited age range, generalizing the efficiency of CoQ10 supplementation to the general population should be done with caution. In this study, due to the limited number of studies with similar health status, subgroup analysis based on health status was not applicable.

Differences in subgroup analyses for dose and duration, regarding TAC and SOD, reflect that the effect of CoQ10 supplementation on inflammation and oxidative stress parameters was neither dose-dependent nor time-dependent. Different CoQ10 formulations with various bioavailability may account for the unclear direct dose-effect or duration-effect relationships observed (Fan et al., 2017). Few studies have shown a more beneficial effect of CoQ10 supplementation on oxidative stress factors over longer periods (>8 weeks) compared to shorter ones (<8 weeks) (Akbari et al., 2020; Hajiluian et al., 2021; Dai et al., 2022). Nevertheless, other studies claimed that the beneficial effects were observed in shorter durations (Farsi et al., 2019). The broad range of CoQ10 supplementation among studies (65–300 mg/day) can also explain the unclear dose-effect relationship between CoQ10 supplementation with TAC and SOD levels. Thus, the optimal dose of CoQ10 supplementation could not be determined precisely for the general population. These discrepancies are mainly related to the small number of studies and participants, as well as the inclusion of healthy population in some studies, which may not be affected by CoQ10 (Fan et al., 2017; Zhai et al., 2017; Mazidi et al., 2018). Although we witnessed a low heterogeneity and publication bias in the reported findings, the results of the present study should be interpreted with caution.

The most notable strength of this umbrella meta-analysis was that it included a considerable number of high-quality methodological studies. Subgroup analyses, controlling publication bias, and conducting a comprehensive systematic search were among the other strengths of our study. However, there were a few limitations that must be noted. First, we were unable to assess the effect of CoQ10 supplementation on other oxidative stress parameters since there were insufficient studies. Second, some studies had been repeated in several meta-analyses. Although this could affect the results, further assessments indicated that repeated studies did not affect the final results. Third, the included studies had been accomplished in certain geographic regions, which may enhance the possibility of selection bias. Fourth, due to the limited number of SMD studies on CRP, subgroup analysis for this biomarker was not possible. Hence, we could not reach a conclusive finding regarding the effect of CoQ10 supplementation on CRP level in different subgroups. Fifth, since most studies did not determine the serum level of CoQ10, they could not ensure that the patients were actually taking their CoQ10 supplements. However, to evaluate the degree of compliance, they used other ways such as assessing the remaining supplements returned by the patients.

## 5 Conclusion

The present umbrella meta-analysis confirmed the potential benefits of CoQ10 supplementation in reducing inflammatory and oxidative stress parameters. Moreover, acute CoQ10 supplementation (≤10 weeks) at doses of >200 mg/day contributed to lower MDA, TNF-α, and IL-6 levels. However, regarding TAC and SOD, over 10 weeks of CoQ10 supplementation with ≤200 mg/day doses resulted in greater increases in TAC and serum SOD activity, respectively. Hence, it appears that the effect of CoQ10 supplementation on inflammatory and oxidative stress can be found in both acute and chronic interventions at low doses and high doses. In this regard, CoQ10 supplementation can be advised as a complementary treatment in chronic inflammatory conditions.

## Data Availability

The original contributions presented in the study are included in the article/Supplementary Material, further inquiries can be directed to the corresponding authors.

## Abbreviations

CoQ10, Coenzyme Q10; WMD, weighted mean difference; SMD, standardized mean difference; CRP, C-reactive protein; MDA, malondialdehyde; TAC, total antioxidant capacity; SOD, superoxide dismutase; TNF- α, tumor-necrosis factor-alpha; IL-6, interleukin-6; ROS, reactive oxygen species; NF-kB, nuclear-factor-kappa-B; DM, diabetes mellitus; T2DM, 2 diabetes mellitus; ES, effect sizes; CIs, confidence intervals; GRADE, Grading of Recommendations, Assessment and Evaluation; miR-146a, microRNAs-146a; IRAK-1, IL-1 receptor-associated kinase-1; TRAF6, TNF receptor-associated factor 6; Nrf2, nuclear factor (erythroid-derived 2)-like 2; HO-1, heme oxygenase 1; PPAR-γ and α, peroxisome proliferator- activated receptor gamma and alpha; BMI, body mass index.
